# Supplementary material for: FastqPuri: high-performance preprocessing of RNA-seq data
Source: BMC Bioinformatics. 2019 May 3;20:226. doi: 10.1186/s12859-019-2799-0 (PMC6500068; doi:10.1186/s12859-019-2799-0)
Supplement: Supplementary file 2 — Archive of FastqPuri. Archive containing all files needed to install and run FastqPuri v1.0.6. Date stamp March 22, 2019. (GZ 47,819 kb) [file 12859_2019_2799_MOESM2_ESM.gz › FastqPuri-1.0.6/html/tree_8h.html]

FastqPuri: include/tree.h File Reference


FastqPuri


- include

Classes |
Typedefs |
Functions

tree.h File Reference

Construction of tree, check paths, write tree, read in tree.
More...

`#include <stdint.h>`  
`#include "defines.h"`  
`#include "fa_read.h"`

Include dependency graph for tree.h:

This graph shows which files directly or indirectly include this file:

Go to the source code of this file.

|  |  |
| --- | --- |
| Classes | |
| struct | \_node |
|  | Node structure: formed out of T\_ACGT pointers to Node structure. More... |
|  | |
| struct | \_tree |
|  | structure containing a T\_ACGT-tree. More... |
|  | |

|  |  |
| --- | --- |
| Typedefs | |
| typedef struct \_node | Node |
|  | Node structure: formed out of T\_ACGT pointers to Node structure. |
|  | |
| typedef struct \_tree | Tree |
|  | structure containing a T\_ACGT-tree. More... |
|  | |

|  |  |
| --- | --- |
| Functions | |
| Node \* | get\_new\_pool (Tree \*tree\_ptr) |
|  | reallocs pool\_2D (++NPOOL\_2D) if all existing nodes have been used More... |
|  | |
| Node \* | new\_node\_buf (Tree \*tree\_ptr) |
|  | moves to the next node (allocating new memory if necessary) More... |
|  | |
| void | free\_all\_nodes (Tree \*tree\_ptr) |
|  | frees the whole tree structure More... |
|  | |
| void | insert\_Lmer (Tree \*tree\_ptr, char \*Lmer) |
|  | Lmer insertion in the tree (depth L). |
|  | |
| void | insert\_entry (Tree \*tree\_ptr, Fa\_entry \*entry) |
|  | fasta entry insertion in the tree (depth L). |
|  | |
| double | check\_path (Tree \*tree\_ptr, char \*read, int Lread) |
|  | checks if read is found in tree and outputs a score More... |
|  | |
| Tree \* | tree\_from\_fasta (Fa\_data \*fasta, int L) |
|  | create Tree structure from fasta structure. More... |
|  | |
| void | save\_tree (Tree \*tree\_ptr, char \*filename) |
|  | saves Tree to disk in filename More... |
|  | |
| Tree \* | read\_tree (char \*filename) |
|  | read tree from file More... |
|  | |

## Detailed Description

Construction of tree, check paths, write tree, read in tree.

Author
:   Paula Perez paula.nosp@m.pere.nosp@m.zrubi.nosp@m.o@gm.nosp@m.ail.c.nosp@m.om

Date
:   18.08.2017

## Typedef Documentation

## ◆ Tree

typedef struct \_tree Tree

structure containing a T\_ACGT-tree.

The tree structure is stored in a pointer to pointer to Node. We grow the structure on the flight as we need more memory. In the outer direction, we start by allocating NPOOL\_2D pointers to Node. In the inner direction, we allocate NPOOL\_1D Nodes and fill them as we read the fasta file. When all of them are allocated, we allocate again NPOOL\_1D. If NPOOL\_2D pointers to Node are allocated, the outer dimension is reallocated with +NPOOL\_2D extra elements. L is the depth of the tree, pool\_count is the number on Node\* elements used so far, pool\_available is the number of Nodes available in every moment, and nnodes is the total number of nodes filled in. We limit the number of allocated nodes to UINT\_MAX (we cannot count more nodes!).

## Function Documentation

## ◆ check\_path()

|  |  |  |  |
| --- | --- | --- | --- |
| double check\_path | ( | Tree \* | *tree\_ptr*, |
|  |  | char \* | *read*, |
|  |  | int | *Lread* |
|  | ) |  |  |

checks if read is found in tree and outputs a score

Parameters
:   |  |  |
    | --- | --- |
    | tree\_ptr | pointer to Tree structure |
    | read | Read or reverse complement |
    | Lread | length of read |

Returns
:   score = (number of Lmers of reads found in read) / (Lread-L+1)

## ◆ free\_all\_nodes()

|  |  |  |  |  |  |
| --- | --- | --- | --- | --- | --- |
| void free\_all\_nodes | ( | Tree \* | *tree\_ptr* | ) |  |

frees the whole tree structure

Parameters
:   |  |  |
    | --- | --- |
    | tree\_ptr | pointer to Tree structure |

This function deallocates the memory allocated in a Tree structure.

## ◆ get\_new\_pool()

|  |  |  |  |  |  |
| --- | --- | --- | --- | --- | --- |
| Node\* get\_new\_pool | ( | Tree \* | *tree\_ptr* | ) |  |

reallocs pool\_2D (++NPOOL\_2D) if all existing nodes have been used

Parameters
:   |  |  |
    | --- | --- |
    | tree\_ptr | pointer to Tree structure |

## ◆ new\_node\_buf()

|  |  |  |  |  |  |
| --- | --- | --- | --- | --- | --- |
| Node\* new\_node\_buf | ( | Tree \* | *tree\_ptr* | ) |  |

moves to the next node (allocating new memory if necessary)

Parameters
:   |  |  |
    | --- | --- |
    | tree\_ptr | pointer to Tree structure |

Returns
:   address to next node

The function checks if there are available nodes (information stored in the variable tree\_ptr -> pool\_available) and goes to the next node. If there is no nodes left, it allocates a new pool\_1D, and if there is no room left in the outter dimension, it reallocates NPOOL\_2D more Node\*'s. If the number of nodes reaches UINT\_MAX, the program returns an error message and exits.

## ◆ read\_tree()

|  |  |  |  |  |  |
| --- | --- | --- | --- | --- | --- |
| Tree\* read\_tree | ( | char \* | *filename* | ) |  |

read tree from file

Parameters
:   |  |  |
    | --- | --- |
    | filename | string with the filename |

Returns
:   pointer to Tree structure

This function unwinds the process carried out in save\_tree and assigns addresses to the children of every given node.

## ◆ save\_tree()

|  |  |  |  |
| --- | --- | --- | --- |
| void save\_tree | ( | Tree \* | *tree\_ptr*, |
|  |  | char \* | *filename* |
|  | ) |  |  |

saves Tree to disk in filename

Parameters
:   |  |  |
    | --- | --- |
    | tree\_ptr | pointer to Tree structure |
    | filename | string containing filename |

The tree structure is stored as follows: every address is stored in a uint32\_t (we are not allowing trees with more than UINT\_MAX nodes). For every node, the addresses of the children are stored in the following fashion:

- If it is pointing to NULL: 0.
- Otherwise: i2, the index in the outer dimension of pool\_2D is identified, and the difference jump = pool\_2D[i][j].children[k] - pool\_2D[i2] is computed. i2\*NPOOL\_D1 + jump is then stored for child k.

## ◆ tree\_from\_fasta()

|  |  |  |  |
| --- | --- | --- | --- |
| Tree\* tree\_from\_fasta | ( | Fa\_data \* | *fasta*, |
|  |  | int | *L* |
|  | ) |  |  |

create Tree structure from fasta structure.

Parameters
:   |  |  |
    | --- | --- |
    | fasta | pointer to fasta structure |
    | L | tree length |


---

Generated on Mon Mar 19 2018 23:42:01 for FastqPuri by  

 1.8.14
